# Pyroptosis: a potential target and research progress of exercise in ameliorating Alzheimer’s disease pathology

**DOI:** 10.3389/fnagi.2026.1983962

**Published:** 2026-10-07

**Authors:** Shao Jie, Zhu Lei

**Affiliations:** School of Physical Education, Qufu Normal University, Qufu, Shandong, China

**Keywords:** Alzheimer’s disease, exercise, inflammasome, neuronal injury, pyroptosis

## Abstract

Alzheimer’s disease (AD), a debilitating neurodegenerative disorder that severely threatens elderly health, is clinically manifested as cognitive decline and driven by core pathological hallmarks including amyloid-beta (Aβ) deposition, tau hyperphosphorylation, and chronic neuroinflammation. Recent studies have indicated that its pathophysiological process is closely associated with pyroptosis, and excessive activation of pyroptosis drives a vicious cycle of amplified inflammation and neuronal injury. Although accumulating evidence suggests that exercise serves as an effective non-pharmacological intervention to suppress pyroptosis and alleviate AD, the intrinsic interactive mechanisms among exercise, pyroptosis and the pathological progression of AD remain incompletely elucidated. Therefore, this review systematically summarizes the functional mechanisms of pyroptosis in the pathological progression of AD and discusses the potential neuroprotective effects of exercise intervention. It further focuses on analyzing the specific mechanisms by which exercise inhibits pyroptosis via regulating the NLRP3 inflammasome pathway, aiming to identify novel targets for the prevention and treatment of AD and provide a theoretical basis for formulating scientific exercise intervention strategies.

## 1 Introduction

Alzheimer’s disease (AD) remains the leading cause of dementia worldwide. Driven by an increasingly aging global population, it is rapidly emerging as one of the most lethal and socioeconomically burdensome diseases of this century. Epidemiological projections even suggest that the global prevalence of dementia could triple by the year 2025 (Gustavsson et al., 2023; Scheltens et al., 2021). The earliest stages of AD are clinically characterized by the cerebral accumulation of two aberrant proteins. The first involves β-amyloid (Aβ) plaques, which are insoluble aggregates forming extracellularly between neurons. The second hallmark is neurofibrillary tangles (NFTs), resulting from the hyperphosphorylation of tau proteins intracellularly, a process that ultimately dismantles the neuronal cytoskeleton (Disouky et al., 2026). Over time, these pathological alterations inflict progressive neuronal damage and subsequent brain atrophy, particularly devastating memory- and cognition-associated regions such as the hippocampus and cerebral cortex (Ogbodo et al., 2022). Abnormally activated cell death pathways exert a cumulative effect, propelling inflammatory cascades forward. Because neuronal loss and cortical shrinkage represent the terminal endpoints of AD pathology, targeting inflammation-mediated neuronal death offers a crucial therapeutic window.

Pyroptosis, a more recently characterized inflammatory form of programmed cell death (PCD) (Rao et al., 2022), acts as a potent driver of this neuronal demise. Mechanistically, it is typically triggered by the activation of inflammasomes—most notably the nucleotide-binding oligomerization domain-like receptor protein 3 (NLRP3)—alongside inflammatory caspases, and is ultimately executed by gasdermin proteins. Biologically, pyroptosis operates as a double-edged sword. Upon invasion by pathogens like bacteria or viruses, host cells recognize pathogen-associated molecular patterns (PAMPs) to activate inflammasomes. This prompts the release of pro-inflammatory cytokines (such as IL-1β and IL-18) and initiates pyroptosis (Broz and Dixit, 2016; Kovacs and Miao, 2017). The resulting cellular swelling and membrane rupture expose the concealed pathogens to immune clearance. Conversely, the chronic, aberrant hyperactivation of pyroptosis—and the ensuing massive release of IL-1β and IL-18—fuels the progression of chronic inflammatory diseases (Liu et al., 2016; Swanson et al., 2019).

In the context of AD, neuroinflammation is a highly complex yet pivotal factor intricately tied to pyroptotic activation and neuronal death. Substantial historical evidence has cemented the inextricable link between pyroptosis and AD (Heneka et al., 2013; Huang et al., 2022; Anderson et al., 2023). Triggered primarily by core AD pathological products (Aβ plaques and Tau tangles), pyroptosis has been identified as a central mechanism amplifying neuroinflammation and exacerbating neuronal loss. This creates a relentless “neuroinflammation-pyroptosis-neuronal damage” vicious cycle, suggesting that curtailing pyroptosis could open new avenues for ameliorating AD pathology (Portela Moreira et al., 2026).

At present, no pharmacological treatment can fundamentally cure Alzheimer’s disease (AD). Cholinesterase inhibitors and N-methyl-D-aspartate (NMDA) receptor antagonists are primarily used to alleviate or maintain patients’ cognitive and clinical symptoms. In recent years, anti-β-amyloid (Aβ) monoclonal antibodies, such as lecanemab and donanemab, have been approved for selected patients with early-stage AD and have been shown to modestly slow cognitive and functional decline (van Dyck et al., 2023; Sims et al., 2023; Cummings et al., 2023). However, their benefits are primarily limited to delaying disease progression rather than reversing or curing the disease, and their use may be associated with safety risks, including amyloid-related imaging abnormalities (ARIA) (Cummings et al., 2023).

Multiple studies have identified physical activity (PA) as one of the major modifiable factors influencing the pathophysiological progression of AD (Livingston et al., 2024). Recent evidence further suggests that exercise may attenuate NLRP3 inflammasome activation, inhibit the activation of caspase-4/5/11 and GSDMD cleavage-mediated pore formation, and consequently suppress pyroptotic signaling and reduce the release of inflammatory cytokines, such as IL-1β and IL-18 (Yu P. et al., 2021). Although the inhibitory effects of exercise on pyroptosis have increasingly been recognized, the precise mechanisms underlying this effect, as well as the complete pathway through which pyroptosis contributes to the initiation and progression of AD pathology, remain to be fully elucidated. Accordingly, this review examines the effects of pyroptosis—an inflammasome-associated proinflammatory form of programmed cell death—on AD-related neurodegeneration. It also discusses how exercise may modulate the expression and activity of key pyroptosis-related molecules, including NLRP3 and GSDMD in the canonical pathway and cytosolic LPS-mediated caspase-4/5/11 in the non-canonical pathway, thereby ameliorating AD-related pathology.

## 2 Pyroptosis

### 2.1 Overview of pyroptosis

The earliest studies of pyroptosis can be traced to a 1986 report by Friedlander, who showed that treatment of mice with anthrax lethal toxin induced cell death accompanied by the rapid release of intracellular contents (Yu P. et al., 2021). In 1989, Cerretti et al. (1992) first identified interleukin-1β-converting enzyme, caspase-1, as an inflammatory caspase (Black et al., 1989). In 1992, Zychlinsky et al. (1992) first described pyroptosis after observing programmed cell death in macrophages infected with the Gram-negative pathogen Shigella flexneri. This form of cell death was initially considered apoptosis because it shared several apoptotic characteristics, including caspase dependence, DNA damage, and nuclear condensation. However, subsequent observations demonstrated that it was distinct from apoptosis.

In Cookson and Brennan (2001) formally defined and coined the term “pyroptosis,” combining the Greek prefix “pyro-” (fire), which reflects its inflammatory nature, with “-ptosis” (falling), consistent with the nomenclature of apoptosis. The term was introduced to distinguish caspase-1-dependent inflammatory programmed cell death from non-inflammatory apoptosis (Cookson and Brennan, 2001). Subsequently, the 2018 definition proposed by the Nomenclature Committee on Cell Death (NCCD) established standardized criteria for the recognition of pyroptosis within the global scientific community (Galluzzi et al., 2018).

### 2.2 Molecular mechanisms of pyroptosis

#### 2.2.1 Canonical pyroptosis pathway

The canonical pyroptosis pathway is primarily mediated by inflammasomes. Its fundamental process can be summarized as follows: after recognizing danger signals, cells initiate inflammasome assembly, which subsequently activates caspase-1, promotes the cleavage of gasdermin D (GSDMD), and induces pore formation in the plasma membrane, ultimately resulting in the release of inflammatory mediators and inflammatory cell death (Broz and Dixit, 2016; Shi et al., 2015; Kayagaki et al., 2015; Liu et al., 2016; Miao et al., 2023). When cells are stimulated by pathogen-associated molecular patterns (PAMPs) or damage-associated molecular patterns (DAMPs), intracellular pattern-recognition receptors are activated first (Man and Kanneganti, 2015; Broz and Dixit, 2016). Among these receptors, the NLRP3 inflammasome is the most extensively studied (Swanson et al., 2019). Other receptors, including AIM2, NLRC4, and NLRP1, can also function as inflammasome sensors and participate in related processes (Swanson et al., 2019).

A key feature of NLRP3 is its broad responsiveness to structurally diverse stimuli, including bacterial toxins, viral components, ATP, urate crystals, cholesterol crystals, silica, amyloid proteins, and disturbances in intracellular ion homeostasis (Swanson et al., 2019; Mariathasan et al., 2006; Hornung et al., 2008; Halle et al., 2008; Duewell et al., 2010). However, these stimuli do not necessarily bind directly to NLRP3. More commonly, they indirectly promote NLRP3 activation by inducing a series of shared cellular stress events, such as potassium (K+) efflux, altered calcium (Ca^2+^) homeostasis, mitochondrial damage, reactive oxygen species production, lysosomal rupture, and disassembly of the trans-Golgi network (Swanson et al., 2019; Hornung et al., 2008; Zhou et al., 2011; Murakami et al., 2012; Muñoz-Planillo et al., 2013; Chen and Chen, 2018). These changes promote the transition of NLRP3 from an autoinhibited state to an activated state capable of oligomerization (Figure 1; He et al., 2016; Sharif et al., 2019).

**FIGURE 1:**
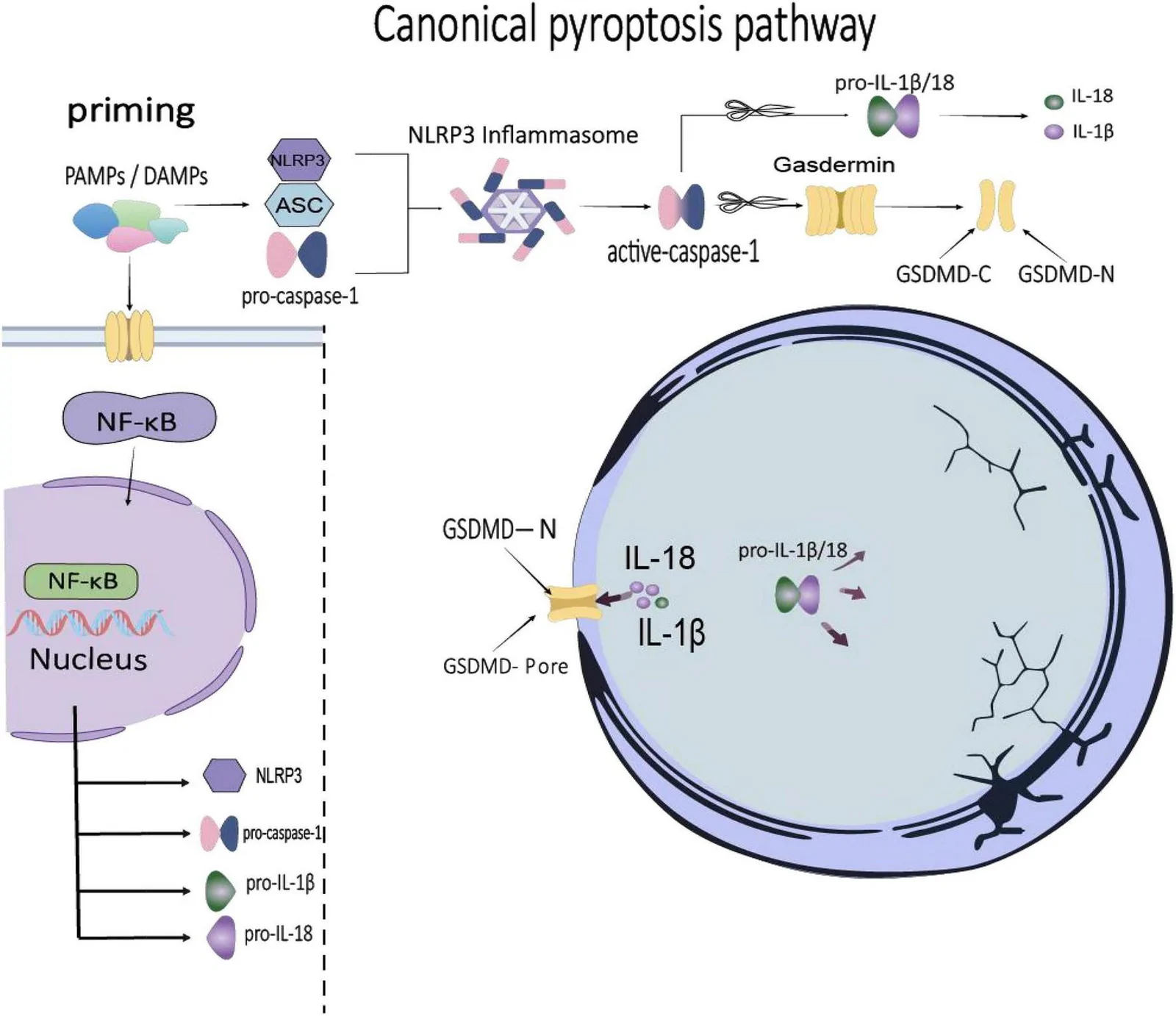
Canonical pyroptosis pathway. Priming, priming signal; PAMPs, pathogen-associated molecular patterns; DAMPs, damage-associated molecular patterns; NF-κB, nuclear factor kappa B; NLRP3, NOD-, LRR-, and pyrin domain-containing protein 3; ASC, apoptosis-associated speck-like protein containing a CARD; pro-caspase-1, the precursor of caspase-1; caspase-1; pro-IL-1β and pro-IL-18, the precursors of interleukin-1β and interleukin-18, respectively; IL-1β and IL-18, mature interleukin-1β and interleukin-18, respectively; GSDMD-N, the N-terminal fragment of gasdermin D; GSDMD-C, the C-terminal fragment of gasdermin D; GSDMD pore, the membrane pore formed by gasdermin D. This figure was created using Adobe Illustrator 2025.

Upon activation, NLRP3 recruits the adaptor protein ASC through pyrin domain (PYD)-PYD interactions. ASC subsequently binds pro-caspase-1 through caspase recruitment domain (CARD)-CARD interactions, thereby forming a large inflammasome complex. This complex serves as a molecular platform that brings multiple pro-caspase-1 molecules into close proximity, enabling their autocatalytic cleavage and activation. The concept of the inflammasome as a molecular platform for the activation of inflammatory caspases and the processing of IL-1β was first proposed by Martinon et al. (2002) in their study of the NLRP1-associated complex, laying the foundation for subsequent investigations into pyroptosis. Inflammasomes were later shown to comprise a group of signaling platforms formed by distinct sensors, ASC, and inflammatory caspases. Among them, the NLRP3 inflammasome has become the most extensively studied molecular complex in the canonical pyroptosis pathway because of its broad stimulus spectrum and close association with both infection and sterile inflammation (Schroder and Tschopp, 2010).

In the canonical pyroptosis pathway, caspase-1 plays a central role. On the one hand, it cleaves inactive pro-IL-1β and pro-IL-18 into their mature forms, IL-1β and IL-18. IL-1β primarily promotes the recruitment of inflammatory cells, increases vascular permeability, and amplifies inflammatory responses, whereas IL-18 is more closely associated with interferon-gamma production and the activation of natural killer cells and T cells. On the other hand, caspase-1 directly cleaves GSDMD, releasing its N-terminal pore-forming domain from the autoinhibitory C-terminal domain. This step is generally considered to mark the transition from upstream signal activation to the execution phase of pyroptosis. Shi et al. (2015), Kayagaki et al. (2015) independently demonstrated that cleavage of GSDMD by inflammatory caspases is a decisive event in pyroptosis. Cells lacking GSDMD cannot undergo typical membrane-rupturing pyroptosis even when upstream inflammasomes and caspase-1 are activated (Shi et al., 2015; Kayagaki et al., 2015).

The N-terminal fragment generated by GSDMD cleavage has a strong affinity for membrane lipids. It recognizes phospholipids enriched on the inner leaflet of the plasma membrane, including phosphoinositides and phosphatidylserine, and subsequently oligomerizes within the membrane to form pores with diameters of several tens of nanometers. Structural and functional studies have further shown that gasdermin family proteins generally exist in an autoinhibited conformation under resting conditions, in which the C-terminal domain suppresses the pore-forming activity of the N-terminal domain. Following proteolytic cleavage and relief of this autoinhibition, the N-terminal fragment inserts into the plasma membrane and assembles into pores. This mechanism represents an important molecular basis distinguishing pyroptosis from conventional apoptosis (Liu et al., 2016; Ding et al., 2016).

As GSDMD pores form, plasma membrane permeability increases rapidly. Potassium ions continuously efflux, whereas sodium ions and water enter the cell in large amounts, ultimately causing cellular swelling. Meanwhile, cytosolic contents, including lactate dehydrogenase, are released, and mature IL-1β and IL-18 can be exported through GSDMD pores or released following plasma membrane rupture (Ding et al., 2026). Consequently, cells progressively lose membrane integrity and exhibit the characteristic features of inflammatory cell death.

#### 2.2.2 Non-canonical pyroptosis pathway

Unlike the canonical pathway, the non-canonical pyroptosis pathway does not initially rely on conventional inflammasome-mediated activation of caspase-1. Instead, it is initiated by LPS-induced activation of inflammatory caspases. Early studies identified murine caspase-11 as a key mediator of non-canonical inflammasome activation and endotoxin-associated inflammatory responses, distinguishing this pathway from the classical caspase-1-dependent inflammasome pathway (Kayagaki et al., 2011). Subsequent studies demonstrated that cytosolic LPS can induce caspase-11 activation, pyroptosis, and endotoxin shock-related responses independently of Toll-like receptor 4 (TLR4), establishing cytosolic LPS as a critical initiating signal in the non-canonical pathway (Hagar et al., 2013; Kayagaki et al., 2013).

Three major mechanisms of LPS transport have been identified. First, outer membrane vesicles (OMVs) released by Gram-negative bacteria can enter host cells through clathrin-mediated endocytosis. Subsequent damage or rupture of the endosomal or vesicular membrane releases LPS into the cytosol, where it binds to and activates caspase-4, caspase-5, or caspase-11, thereby initiating the noncanonical pyroptosis pathway (Chen et al., 2018). Second, during intracellular bacterial infection, interferon-inducible guanylate-binding proteins (GBPs) are recruited to the bacterial surface and bacteria-containing vacuoles, where they disrupt the integrity of the bacterial envelope and the surrounding vacuolar membrane. This promotes the exposure and cytosolic release of bacterial LPS, thereby enhancing caspase-11-dependent inflammasome activation and pyroptosis (Santos et al., 2018). Finally, under systemic inflammatory conditions such as sepsis, high-mobility group box 1 (HMGB1) can function as an LPS delivery protein by forming complexes with LPS and mediating their internalization through the receptor for advanced glycation end products (RAGE). After entering the cell, HMGB1–LPS complexes dissociate within acidic endosomal or lysosome-associated compartments and facilitate the translocation of LPS into the cytosol, ultimately inducing caspase-11 activation and sepsis-associated inflammatory responses (Deng et al., 2018).

Thus, caspase-4/5/11 function not only as downstream effector enzymes but also as direct intracellular sensors of LPS (Shi et al., 2014). Once activated, these inflammatory caspases cleave GSDMD and release its pore-forming N-terminal fragment. The GSDMD N-terminal fragment subsequently oligomerizes in the plasma membrane to form pores, resulting in increased membrane permeability, disruption of ion homeostasis, cellular swelling, and ultimately pyroptotic cell death (Kayagaki et al., 2015).

In addition, GSDMD pore formation promotes K+ efflux, which can secondarily activate the NLRP3 inflammasome. NLRP3 activation then promotes caspase-1-dependent maturation and release of IL-1β and IL-18. In this manner, the non-canonical pathway is linked to the canonical NLRP3–caspase-1 axis, thereby amplifying the inflammatory response (Rühl and Broz, 2015).

#### 2.2.3 Caspase-3-related pyroptotic signaling

The caspase-3/GSDMD-related mechanism, which has recently attracted increasing attention, should be interpreted with caution. Caspase-3 is traditionally recognized as a key executioner caspase in apoptosis. To date, its more clearly established and better-supported pyroptosis-related substrate is GSDME rather than GSDMD. During apoptosis, caspase-3 can cleave GSDME and release its pore-forming N-terminal fragment (Ouyang et al., 2024). This process may convert cell death that would otherwise exhibit apoptotic features into pyroptosis-like death characterized by plasma membrane pore formation, cellular swelling, and the release of inflammatory intracellular contents (D’Alessandro et al., 2025).

In contrast, although some studies have suggested that caspase-3/7 can cleave GSDMD at specific sites under apoptosis-related conditions, this cleavage is not equivalent to the pyroptosis-inducing activation of GSDMD mediated by caspase-1 or caspase-4/5/11. Instead, it may attenuate or restrict the pore-forming activity of the GSDMD N-terminal fragment. Therefore, this mechanism is more appropriately viewed as a form of crosstalk between apoptosis and pyroptosis, rather than as a well-established major pyroptotic pathway comparable to the canonical or non-canonical pathways (Taabazuing et al., 2017).

## 3 Pyroptosis and the pathological progression of Alzheimer’s disease

### 3.1 Evidence for pyroptosis activation in patients with Alzheimer’s disease and animal models

Evidence from both patient samples and experimental animal models indicates that pyroptosis-related pathways are activated in Alzheimer’s disease (AD). Saresella et al. (2016) examined peripheral blood mononuclear cells from patients with AD and reported markedly increased expression of NLRP3 and NLRP1 inflammasomes, caspase-1, and interleukin-1β-related signaling molecules, suggesting activation of inflammasome pathways in these patients. Using Aβ-treated neurons and AD model systems, Tang (2025) found that Aβ induced the activation of NLRP1 and caspase-1. Inhibition of NLRP1 attenuated neuronal injury, indicating that Aβ may induce NLRP1-dependent pyroptosis in neurons (Tang, 2025).

Further evidence was provided by Heneka et al. (2013) in APP/PS1 mice. Deletion of either NLRP3 or caspase-1 reduced Aβ deposition, alleviated microglia-mediated inflammatory responses, and improved cognitive performance, supporting a role for the NLRP3/caspase-1 axis in AD progression. In a study using 5xFAD mice, Li L. et al. (2025) reported that exercise training significantly decreased the cerebral expression of NLRP3, ASC, and caspase-1 and suppressed the activation of GSDMD, a key executor of pyroptosis. These findings suggest that exercise may improve anxiety-like behavior and cognitive impairment in AD mice by inhibiting NLRP3-mediated microglial pyroptosis (Li L. et al., 2025).

Taken together, findings from patient-derived samples, Aβ-induced cellular models, and APP/PS1 animal models consistently indicate aberrant activation of inflammasome–caspase-1 signaling in AD. Pyroptosis may therefore represent more than a secondary feature of AD pathology and could contribute to neuroinflammation, Aβ accumulation, and cognitive decline.

### 3.2 A new perspective on hallmark features of AD and pyroptosis

The major pathological hallmarks of Alzheimer’s disease include abnormal β-amyloid (Aβ) deposition, tau hyperphosphorylation, neurofibrillary tangle formation, impaired synaptic structure and function, and progressive neuronal loss. Growing insight into the mechanisms of neuroinflammation has suggested that these pathological changes not only contribute directly to neuronal injury but may also act as endogenous danger signals. By sustaining inflammatory activation in the brain, they may further promote pyroptotic cell death (Jack et al., 2024; Sims et al., 2023).

#### 3.2.1 Mechanisms by which Aβ induces inflammasome activation and pyroptosis

In the AD brain, β-amyloid (Aβ) oligomers and fibrillar deposits can be recognized by microglia as damage-associated signals, thereby inducing microglial activation. Halle et al. (2008) showed that Aβ acts as an endogenous danger signal capable of activating the NLRP3 inflammasome in microglia. This finding suggests that inflammasome signaling may represent an important mechanism underlying Aβ-induced neuroinflammation. Following activation, microglia can upregulate a range of inflammation-related molecules through nuclear factor-κB (NF-κB) signaling, including NLRP3, pro-IL-1β, and pro-IL-18 (Moonen et al., 2023). This process is commonly regarded as the “priming” step of inflammasome activation and pyroptosis, providing the basis for subsequent amplification of inflammatory cascades (Heneka et al., 2013; Saresella et al., 2016).

Aβ may promote inflammasome activation through several mechanisms. After being internalized by microglia, Aβ can impair lysosomal function and compromise lysosomal membrane integrity, leading to the release of lysosomal contents, such as cathepsins, into the cytoplasm and thereby promoting NLRP3 inflammasome assembly (Halle et al., 2008). Aβ can also induce mitochondrial damage, increase reactive oxygen species production, and cause potassium efflux and disrupted intracellular calcium homeostasis. These intracellular stress signals are important triggers of inflammasome activation and can promote caspase-1 activation and downstream inflammatory responses (Heneka et al., 2013; Kelley et al., 2019). In AD animal models, Heneka et al. (2013) further demonstrated that the NLRP3 inflammasome is aberrantly activated in the brain. Inhibition of this pathway attenuated inflammatory responses and Aβ-associated pathological changes, highlighting the importance of inflammasome activation in Aβ-induced neuroinflammation and pyroptosis.

#### 3.2.2 Pyroptosis exacerbates Aβ pathology through a feed-forward mechanism

Importantly, Aβ-induced pyroptosis is not an isolated form of cell death but is closely linked to the progression of AD pathology through mutually reinforcing processes (Heneka et al., 2013; Halle et al., 2008). Following inflammasome activation, caspase-1 promotes the maturation and release of IL-1β and IL-18. IL-1β may enhance amyloid precursor protein (APP) processing and Aβ production, while also amplifying neuroinflammation through the NLRP3/IL-1β/IL-1R1 axis (Lopez-Rodriguez et al., 2021; Li X. et al., 2025). Together with IL-1β, IL-18 may regulate APP, β-site APP-cleaving enzyme 1 (BACE1), and γ-secretase, thereby influencing Aβ metabolism (Maurya et al., 2025). In addition to their direct effects on Aβ-related pathways, both cytokines can activate downstream signaling cascades, including NF-κB, c-Jun N-terminal kinase (JNK), and p38 mitogen-activated protein kinase (p38 MAPK), through their respective receptors. Persistent activation of these pathways sustains inflammatory signaling and may further promote AD progression (Huang et al., 2025).

Meanwhile, caspase-1-mediated GSDMD cleavage induces plasma membrane pore formation and ultimately triggers pyroptosis (Shi et al., 2015). As pyroptotic cells rupture, large amounts of inflammatory mediators and intracellular contents are released into the extracellular environment. This process disrupts homeostasis in the brain microenvironment and strongly activates neighboring glial cells, resulting in a locally amplified inflammatory response (Heneka et al., 2015). ASC specks formed during inflammasome activation can also be released extracellularly. These specks may serve as molecular scaffolds that directly bind Aβ and accelerate its aggregation and deposition (Venegas et al., 2017).

Thus, pyroptosis should not be considered merely a passive consequence of pathological stimulation. Aβ can trigger pyroptosis, whereas pyroptosis-derived inflammatory mediators and extracellular ASC specks can, in turn, promote further pathogenic protein aggregation. Together, these processes form a self-amplifying cycle in the AD brain that may progressively aggravate disease pathology (Figure 2; Venegas et al., 2017; Heneka et al., 2015).

**FIGURE 2:**
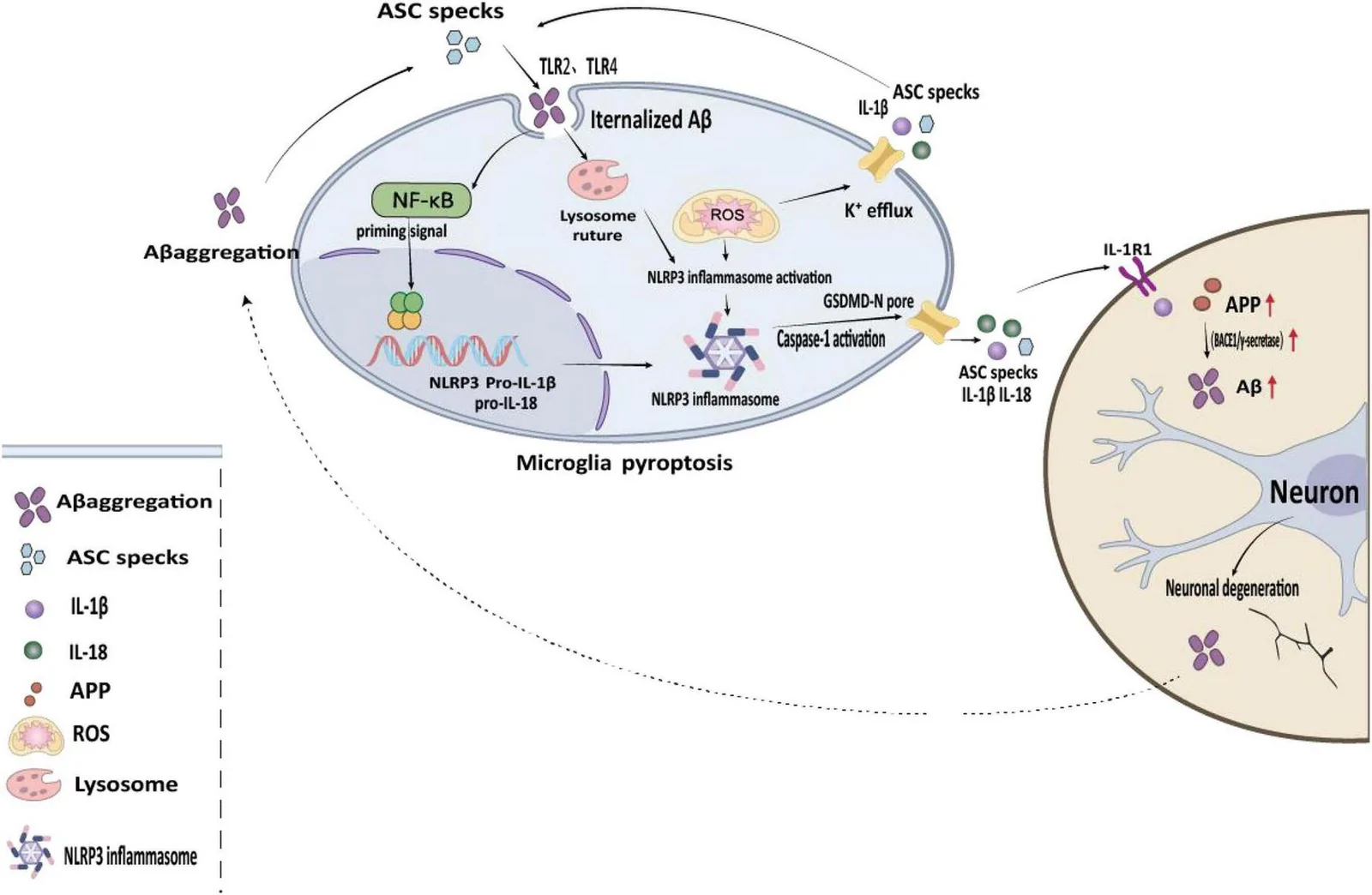
The vicious cycle between pyroptosis and neurons. Aβ aggregation, amyloid-β aggregation; ASC specks, apoptosis-associated speck-like protein containing a CARD (ASC) specks; IL-1β, interleukin-1β; IL-18, interleukin-18; APP, amyloid precursor protein; ROS, reactive oxygen species; lysosome; NLRP3 inflammasome, NOD-, LRR-, and pyrin domain-containing protein 3 (NLRP3) inflammasome; TLR2, Toll-like receptor 2; TLR4, Toll-like receptor 4; internalized Aβ, internalized amyloid-β; NF-κB, nuclear factor kappa B; priming signal; lysosomal rupture; K^+^ efflux, potassium ion efflux; NLRP3 inflammasome activation, activation of the NOD-, LRR-, and pyrin domain-containing protein 3 inflammasome; pro-IL-1β and pro-IL-18, precursor forms of interleukin-1β and interleukin-18, respectively; caspase-1 activation; GSDMD-N pore, pore formed by the N-terminal fragment of gasdermin D. This figure was created using Adobe Illustrator 2025.

#### 3.2.3 Synergistic interactions between tau pathology and pyroptosis

Tau hyperphosphorylation and pyroptosis are not independent pathogenic processes; rather, they are closely intertwined and may reinforce one another in a self-amplifying cycle. Hyperphosphorylated tau (p-tau) and neurofibrillary tangles formed by tau aggregates can act as damage-associated molecular patterns (DAMPs). When microglia internalize these pathological tau aggregates, phagolysosomal instability and membrane rupture may occur, resulting in the release of cathepsin B into the cytoplasm. Together with the accumulation of mitochondrial reactive oxygen species (ROS) induced by tau toxicity, these events provide potent signals for NLRP3 inflammasome assembly (Ising et al., 2019; Stancu et al., 2019). Recent studies have also suggested that internalized tau can further enhance the priming phase of inflammatory transcription through pathways such as cGAS–STING, rendering cells more susceptible to subsequent pyroptotic cascades (Jin et al., 2021).

Following inflammasome activation, caspase-1 cleaves gasdermin D (GSDMD), leading to plasma membrane pore formation and pyroptotic cell death. Large amounts of interleukin-1β (IL-1β) and IL-18 are subsequently released into the extracellular microenvironment. At this stage, a feed-forward effect may emerge. Extracellular IL-1β can bind to receptors on neurons and activate downstream kinases, including glycogen synthase kinase-3β (GSK-3β) and cyclin-dependent kinase 5 (CDK5). Aberrant activation of these kinases promotes further tau hyperphosphorylation, accelerates microtubule destabilization, and impairs axonal transport (Ising et al., 2019; Kitazawa et al., 2005; Ghosh et al., 2013).

### 3.3 Targeting pyroptosis to interrupt pathological feedback loops

Overall, pyroptosis appears to act as an important mechanistic link between Aβ and tau pathology in AD. As an early pathological trigger, Aβ accumulation can induce inflammasome activation and pyroptosis, thereby establishing a chronic neuroinflammatory microenvironment. This inflammatory state may impair microglial Aβ clearance while promoting tau hyperphosphorylation and pathological spread through the regulation of relevant kinases. Accordingly, targeting the NLRP3/caspase-1/GSDMD pyroptosis pathway to limit excessive neuroinflammation and inflammatory cell death may represent a promising strategy for slowing AD progression and improving cognitive function.

## 4 Exercise-mediated inhibition of pyroptosis

### 4.1 Direct inhibitory effects of exercise on NLRP3 inflammasome assembly and activation

Exercise-induced metabolic remodeling appears to play an important role in suppressing NLRP3 inflammasome activity. As exercise intensity increases or exercise duration is prolonged, specific metabolic intermediates released by the liver and skeletal muscle may exert direct immunomodulatory effects. Youm et al. (2015) reported that circulating β-hydroxybutyrate (BHB), whose concentration increases substantially under certain metabolic conditions, can inhibit potassium efflux. This effect may interfere directly with NLRP3 oligomerization and apoptosis-associated speck-like protein containing a CARD (ASC) assembly without relying on conventional receptor-mediated pathways (Youm et al., 2015; Quiroga et al., 2020).

Similarly, lactate produced by skeletal muscle during exercise may function as a signaling molecule. By binding to receptors such as GPR81 on target cells, lactate may reduce caspase-1 cleavage and thereby limit inflammasome maturation at the protein level (Hoque et al., 2014; Brooks, 2018). In addition to the direct actions of metabolites, exercise-related changes in cellular energy status may also restrain NLRP3 activation. ATP consumption during exercise increases the intracellular AMP/ATP ratio, generating an energy-stress signal that activates adenosine monophosphate-activated protein kinase (AMPK) (Herzig and Shaw, 2018). Beyond its role in metabolic regulation, activated AMPK may inhibit the assembly of the NLRP3 inflammasome through substrate phosphorylation and related mechanisms (Trstenjak-Prebanda et al., 2023).

Maintenance of intracellular homeostasis is likewise essential for preventing excessive NLRP3 activation. Exercise is a physiological inducer of autophagy and can increase basal autophagic flux (He et al., 2012). Enhanced autophagy and mitophagy facilitate the removal of dysfunctional organelles and damage-associated molecular patterns (DAMPs), thereby reducing upstream stimuli that promote inflammasome assembly (Zhong et al., 2016). Thus, through the production of anti-inflammatory metabolites, modulation of energy-sensing pathways, and enhancement of autophagic clearance, exercise may exert multi-level inhibitory effects on the NLRP3 inflammasome.

### 4.2 Exercise blocks pyroptosis initiation by regulating upstream signals: ROS, NF-κB, and P2 × 7 receptors

Pyroptosis depends largely on both priming and activation signals, and accumulating evidence indicates that exercise may interrupt this cascade at an early stage. Excessive intracellular reactive oxygen species (ROS) are important initiators of pyroptosis (Fu and Wu, 2023). Through a hormetic response, exercise can activate the nuclear factor erythroid 2-related factor 2/antioxidant response element (Nrf2/ARE) pathway and promote compensatory increases in endogenous antioxidant enzymes, including superoxide dismutase (SOD) and catalase (CAT) (Done and Traustadóttir, 2016). This adaptive response facilitates the removal of excessive mitochondrial ROS, prevents the dissociation of thioredoxin-interacting protein (TXNIP) from thioredoxin, and reduces TXNIP binding to NLRP3. In this way, exercise may disrupt an early trigger for inflammasome activation and pyroptosis (Chien et al., 2016).

At the transcriptional level, nuclear factor-κB (NF-κB) is a key regulator of NLRP3 and pro-inflammatory cytokine precursor expression. Myokine-derived IL-6, which is released during muscle contraction, may exert systemic anti-inflammatory effects by inhibiting the IKK/IκBα phosphorylation cascade and limiting nuclear translocation of the NF-κB p65 subunit (Severinsen and Pedersen, 2020). Restricted p65 nuclear translocation subsequently suppresses the transcription of NLRP3, pro-IL-1β, and pro-IL-18, thereby negatively regulating the priming stage of pyroptosis at the gene-expression level (Pedersen, 2017).

In addition, extracellular ATP accumulation and activation of the P2 × 7 receptor (P2 × 7R) are important events that promote potassium (K^+^) efflux and inflammasome assembly. Long-term exercise has been reported to increase the activity of the ectonucleotidases CD39 and CD73, accelerating the hydrolysis of extracellular ATP to adenosine and reducing ATP concentrations in the local microenvironment. Exercise may also decrease P2 × 7R protein expression in target tissues. By simultaneously reducing both extracellular ATP and P2 × 7R availability, exercise may inhibit ATP–P2 × 7R-mediated K^+^ efflux, disrupt the ionic conditions required for NLRP3 oligomerization, and consequently limit caspase-1 activation and GSDMD cleavage (Di Virgilio et al., 2017).

### 4.3 Protection of GSDMD processing and plasma membrane integrity by exercise

Gasdermin D (GSDMD) is the terminal effector of pyroptosis. Its specific cleavage and the subsequent formation of transmembrane pores are decisive steps leading to plasma membrane rupture (Shi et al., 2015; Broz and Dixit, 2016). The protective effects of exercise may involve both upstream inhibition of GSDMD activation and downstream preservation of membrane integrity.

As described above, regular long-term exercise can reshape the systemic inflammatory milieu, partly by reducing excessive ROS accumulation. These changes may suppress NLRP3 inflammasome activation and caspase-1 activity in affected tissues. Attenuation of these upstream signals limits the cleavage of full-length GSDMD into the pore-forming N-terminal fragment (GSDMD-N), allowing most GSDMD to remain in its inactive, autoinhibited full-length form in the cytoplasm.

At the level of membrane repair, cells adapted to exercise may be better equipped to respond when a limited number of GSDMD pores are formed under local stress conditions. Endosomal sorting complex required for transport III (ESCRT-III)-mediated membrane repair is considered an important mechanism for limiting the lytic outcome of pyroptosis. The ESCRT-III complex promotes outward membrane budding and the shedding of microvesicles containing GSDMD pores, thereby restoring membrane integrity before irreversible cell lysis occurs (Rühl et al., 2018; Zhu et al., 2024). However, current evidence supports only the role of ESCRT-dependent membrane repair in counteracting GSDMD pore-induced damage; it does not directly demonstrate that exercise enhances ESCRT-III-mediated repair. Future studies should therefore investigate whether exercise can facilitate the repair of GSDMD pores at the final stage of pyroptosis (Figure 3).

**FIGURE 3:**
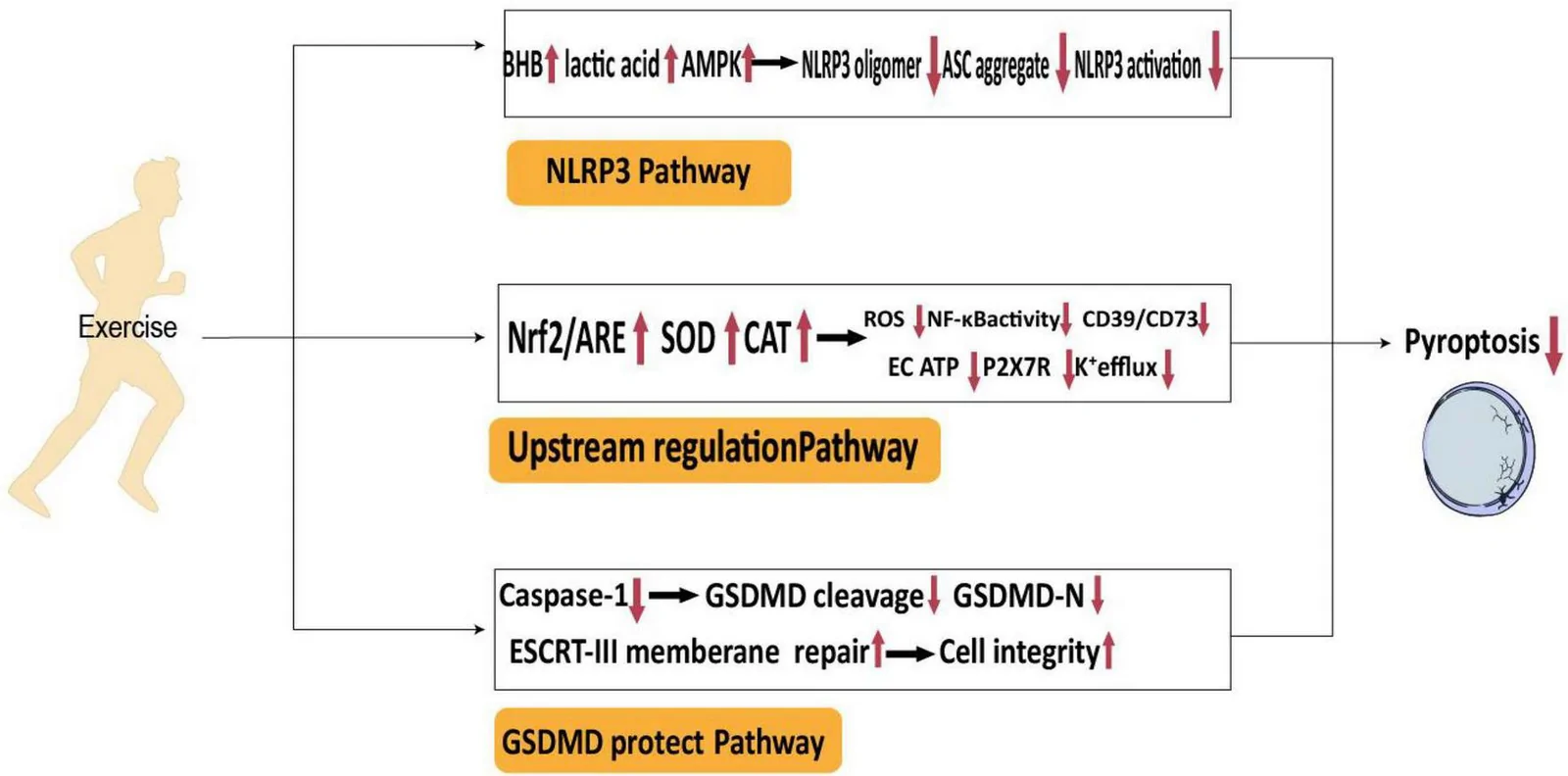
Core pathway of exercise-mediated inhibition of pyroptosis. “↑” indicates promotion, whereas “↓” indicates inhibition. Exercise; pyroptosis; BHB, ß-hydroxybutyrate; AMPK, AMP-activated protein kinase; Nrf2/ARE, nuclear factor erythroid 2-related factor 2/antioxidant response element; SOD, superoxide dismutase; CAT, catalase; ROS, reactive oxygen species; NF-κB, nuclear factor kappa B; CD39/CD73, extracellular nucleotidases; ATP, adenosine triphosphate; P2X7R, P2X7 receptor; K^+^, potassium ions; caspase-1, cysteine-aspartic protease-1; GSDMD, gasdermin D; ESCRT-III, endosomal sorting complexes required for transport III. This figure was created using Adobe Illustrator 2025.

### 4.4 Effects and potential mechanisms of different exercise modalities in regulating pyroptosis and improving AD

#### 4.4.1 Aerobic exercise

Among the various exercise interventions, aerobic exercise is considered an important non-pharmacological strategy for slowing AD progression, partly because of its beneficial effects on systemic metabolism. In contrast to short-duration, high-intensity exhaustive exercise, long-term, regular aerobic exercise at low to moderate intensity—including treadmill running, swimming, cycling, and voluntary wheel running—appears to exert more consistent regulatory effects on neuroinflammation and pyroptosis (Yan et al., 2024; Li N. et al., 2023).

Studies have shown that aerobic exercise can reduce the expression of ROS, TNF-α, IL-18, NF-κB p65, and components of the NLRP3 inflammasome (Zhou et al., 2022). These findings suggest that exercise may modulate inflammasome-related signaling cascades and alleviate AD-associated pathological alterations (Zhou et al., 2022). Wang et al. (2019) observed that aerobic exercise inhibited forkhead box protein O1 (FOXO1) acetylation in the brains of AD rats while promoting FOXO1 phosphorylation. These changes were accompanied by reduced expression of NF-κB and NLRP3 inflammasome-associated proteins. The resulting attenuation of inflammatory responses suggests that exercise may exert anti-inflammatory effects through the FOXO1/NF-κB/NLRP3 inflammasome pathway (Wang et al., 2019).

Liu et al. (2026) demonstrated that lactate, a product of aerobic glycolysis, serves as a critical metabolic signal driving NLRP3 inflammasome activation. They found that macrophages undergo metabolic reprogramming during pyroptosis, which boosts aerobic glycolysis and lactate accumulation. Endogenous lactate directly targets the K24 and K565 sites of the NLRP3 protein via AARS2-dependent lactylation modification. This process facilitates ASC recruitment and inflammasome assembly, ultimately triggering Caspase-1 activation, GSDMD cleavage and IL-1β release. Notably, Liu et al. (2026) further verified that pharmacological inhibition of LDHA to curb lactate production, or blockade of lactate uptake with MCT1 inhibitors, markedly attenuates NLRP3 inflammasome activation and subsequent pyroptosis. This offers a bidirectional perspective on how exercise modulates pyroptosis: while strenuous exercise may cause local lactate accumulation and theoretically trigger pyroptosis, regular moderate exercise [as shown in Zhang’s et al. (2023) study] suppresses NLRP3-mediated pyroptosis by improving systemic metabolic homeostasis and alleviating pathological hyperlactatemia and chronic inflammation (Liu et al., 2026). Furthermore, although Yu F. et al.’s (2021) study on Alzheimer’s disease did not directly measure pyroptosis markers, its results demonstrated that the aerobic cycling group exhibited milder cognitive decline compared with the natural disease progression. This indicates that systemic anti-inflammatory and metabolic improvements induced by exercise may broadly shield neurons from multiple pathological injuries including pyroptosis (Yu F. et al., 2021).

Collectively, aerobic exercise confers neuroprotection against AD progression through the optimization of whole-body metabolic homeostasis and reduction of chronic inflammation. Sustained regular low-to-moderate aerobic training downregulates ROS, TNF-α, IL-18, NF-κB p65, and NLRP3 inflammasome-related proteins, and is capable of curbing inflammation by tuning the FOXO1/NF-κB/NLRP3 signaling axis. Since lactate metabolism strongly correlates with NLRP3 inflammasome activation and pyroptosis, appropriate aerobic exercise can alleviate NLRP3-driven inflammation and pyroptosis risk by correcting lactate metabolism and reversing pathological lactate buildup and metabolic disorders. In addition, aerobic exercise-elicited anti-inflammatory and metabolic improvements can slow AD-related cognitive impairment (Salo et al., 1991; Ding et al., 2024).

#### 4.4.2 Resistance exercise

At present, no consensus has been reached regarding the effects of anaerobic exercise on pyroptosis, as outcomes may vary according to exercise modality, intensity, and duration. Nevertheless, existing evidence indicates that 8 weeks of resistance training can improve the local inflammatory microenvironment and reduce NF-κB expression. This reduction may limit the caspase-1 cleavage cascade and interrupt the execution of pyroptosis, thereby exerting protective effects at the cellular level (Sardeli et al., 2018; Gong et al., 2022).

Another study reported that 12 weeks of resistance training reduced hippocampal expression of NF-κB and the NLRP3 inflammasome in mice with insulin resistance. Resistance training also decreased levels of pyroptosis- and inflammation-related molecules, including GSDMD, GSDMD-N, IL-1β, and IL-18. These findings suggest that resistance training may reduce pyroptosis in the hippocampus by suppressing NF-κB/NLRP3 signaling, thereby providing protection against insulin resistance-associated central inflammatory injury (Ji et al., 2020). Zhang’s et al. (2023), in a randomized controlled trial involving older adults with prediabetes, found that 6 months of Yijinjing combined with resistance training significantly reduced the protein expression of key components of the NLRP3 inflammasome (NLRP3, Cleaved Caspase-1, Cleaved IL-1β) in peripheral blood mononuclear cells (PBMCs), and lowered the concentration of mature IL-1β in serum. Since the NLRP3-Caspase-1-GSDMD axis is the canonical execution pathway of pyroptosis, and robust release of IL-1β constitutes one of the major consequences of pyroptosis, these findings suggest that exercise may block downstream pyroptotic progression by inhibiting aberrant activation of this upstream signaling node.

However, experimental evidence specifically addressing the relationship among resistance exercise, pyroptosis, and AD remains limited. The influences of exercise intensity, frequency, and baseline individual characteristics also require further clarification. Future research should identify the molecular mechanisms through which different resistance-training protocols regulate pyroptosis, thereby supporting the development of more precise exercise-based interventions.

#### 4.4.3 Hypoxic exercise

In recent years, increasing attention has been directed toward the potential of hypoxic exercise to inhibit NLRP3 inflammasome signaling. However, conflicting evidence has also emerged. Li N. et al. (2023) proposed that chronic intermittent hypoxia promotes cardiomyocyte pyroptosis in rats with obstructive sleep apnea–hypopnea syndrome through activation of the canonical NLRP3/caspase-1/GSDMD pathway. This process was characterized by impaired cardiomyocyte membrane integrity, membrane blebbing, and the release of pyroptotic bodies, accompanied by increased expression of caspase-1, GSDMD, GSDMD-N, and NLRP3. These findings indicate that repeated hypoxia/reoxygenation is not merely an adaptive stimulus but may contribute to myocardial inflammatory injury and cardiovascular complications (Li N. et al., 2023).

In contrast, Pang (2024) reported that moderate hypoxic preconditioning exerted protective effects in cerebral ischemia–reperfusion injury. It reduced the expression of NLRP3, caspase-1, GSDMD, and inflammatory cytokines, including IL-1β, IL-6, and IL-8. These changes were associated with improved neurological function, smaller cerebral infarct volumes, less histopathological damage, and reduced cell death in mice (Pang, 2024). Thus, hypoxia does not inevitably promote pyroptosis; its effects may depend on the intensity and duration of hypoxic exposure, as well as whether adaptive preconditioning occurs.

Although Rossetti et al. (2017) did not directly assess pyroptosis-related markers, they found that dietary nitrate supplementation during hypoxic exercise neither alleviated acute mountain sickness nor improved exercise performance. Instead, it increased headache severity, acute mountain sickness severity, and perceived exertion. These findings suggest that enhancing vasodilation and oxygen delivery through nitric oxide-related pathways during hypoxic exercise may not always be beneficial and may even aggravate hypoxia-related discomfort and inflammatory stress (Rossetti et al., 2017).

Overall, hypoxic exercise may have a dual effect on pyroptosis. Moderate, short-term, and carefully controlled hypoxic exercise or hypoxic preconditioning may enhance antioxidant defenses, improve mitochondrial quality control, and inhibit NLRP3 inflammasome activation, thereby reducing pyroptosis and protecting tissues. Conversely, chronic, severe, repetitive, or poorly adapted hypoxic exercise may increase ROS production, mitochondrial dysfunction, Ca^2+^ overload, K^+^ efflux, and inflammatory cytokine release. These events may activate the NLRP3/caspase-1/GSDMD pathway and ultimately promote pyroptosis and tissue injury. Therefore, whether hypoxic exercise suppresses or exacerbates pyroptosis is determined not by hypoxia alone, but by hypoxic dose, exercise intensity, exposure duration, physiological adaptation, and target-organ susceptibility. The NLRP3 inflammasome may serve as a central molecular hub linking hypoxic exercise, inflammation, and pyroptosis (Table 1).

**TABLE 1:** Previous studies on different exercise modalities.

References	Year	Subjects	Exercise modality	Intervention duration	Exercise protocol	Main findings
Rosa et al., 2021	2020	Male Swiss mice	Aerobic exercise training (AET)	4 weeks	Five sessions per week (Monday to Friday), 40 min per session.	Significantly prevented Aβ1–40-induced activation of Iba-1, TXNIP, and the NLRP3 pathway. It also reduced basal TXNIP and caspase-1 levels in vehicle-treated mice.
Wang et al., 2025	2025	7-month-old male APP/PS1 double-transgenic AD mice	AET	8 weeks, 5 days/week	A 5-min warm-up at 5 m/min, followed by 5 min at 8 m/min and 30 min of treadmill running at 12 m/min.	Significantly improved cognitive function, reduced neuronal damage in the hippocampal CA1 region, inhibited NLRP3 inflammasome activation, and decreased cleaved caspase-1 and GSDMD-N expression. Exercise also reduced the release of IL-1β and IL-18.
Zhang, 2022	2022	6-month-old male APP/PS1 transgenic mice	High-intensity interval training (HIIT)	8 weeks, every other day	Treadmill training for 41.5 min per session, including a 5-min warm-up at 40% maximal speed (Smax) followed by nine cycles of 1.5 min running at 85% Smax and 2 min active recovery at 45% Smax.	Significantly improved spatial learning and memory, alleviated hippocampal neuronal injury, reduced IL-1β levels in serum and the hippocampus, and downregulated hippocampal GSDMD and GSDMD-N expression.
Liang et al., 2020	2020	Male APPswe/PS1dE9 (APP/PS1) transgenic AD mice	HIIT and moderate-intensity continuous training (MICT)	12 weeks, 5 days/week	HIIT: **5-**min warm-up at 40% Smax, followed by nine cycles of 1.5 min running at 85% Smax. MICT: Continuous running at 60% Smax, with the total running distance matched to that of the HIIT group. Both protocols included 5-min warm-up and cool-down periods at 40% Smax.	Both HIIT and MICT inhibited NLRP3 inflammasome activation, upregulated phagocytic receptor expression in microglia, promoted Aβ clearance, and reduced pathological Aβ deposition. HIIT was as effective as MICT in improving AD-related pathology.
Gomes da Silva et al., 2013	2013	18-month-old male Wistar rats	AET	10 consecutive days	Adaptation period: 3 days of treadmill exposure for 5 min/day at 8 m/min and 0% incline. Training period: One session daily, beginning at 12 m/min for 10 min and progressively increasing to 15 m/min for 30 min.	Short-term aerobic exercise induced anti-inflammatory effects in the hippocampus of aged rats, primarily reflected by increased IL-10 levels and an improved pro-inflammatory/anti-inflammatory cytokine balance.
Han et al., 2025	2025	SAMP8 mice; C57BL/6J mice receiving hippocampal Aβ1–42 injections; and 5xFAD transgenic mice	AET	8 weeks	Adaptation period: 3 days, with treadmill speed gradually increased to 15 m/min. Training period: 5 days/week, 40 min/day, at a 5° incline; the initial speed was 15 m/min and was increased by 11 m/min weekly to raise exercise intensity.	Lactate was identified as a key mediator linking exercise to synaptic protection and cognitive improvement in AD models. Exercise increased lactate levels and activated the expression of synapse-related genes, thereby improving neurological function; these effects depended on efficient lactate transport.
Ji et al., 2020	2020	6-week-old male C57BL/6J mice with insulin resistance	Resistance training (RT)	12 weeks	Weighted ladder-climbing exercise on a 1-m ladder inclined at 85°, with 2-cm rung spacing. The initial load was 30% of body weight and increased by 10% per week during the first 4 weeks, reaching 80% of body weight in week 5; thereafter, the load increased by 10% every 2 weeks. Training was performed 3 days/week, with three sets of five climbs per day; rest intervals were 2 min between sets and 1 min between climbs.	12 weeks of resistance training effectively inhibited NLRP3 inflammasome activation, alleviated hippocampal pyroptosis and inflammatory responses, and improved hippocampal function in insulin-resistant mice.
Gomarasca et al., 2022	2022	Older women (*n* = 29; mean age, 68 ± 8 years)	Nordic walking	12 weeks	Three sessions per week, each lasting 60 min: 10 min of warm-up, 40 min of Nordic walking, and 10 min of cool-down. Exercise intensity was maintained at 60%–70% of maximal heart rate.	After 12 weeks of training, pre-exercise expression of NLRP3 and TLR4 genes at the end of the intervention was approximately 2-fold lower than baseline. However, a single acute exercise bout at the end of training significantly increased NLRP3 and TLR4 gene expression by 2.6-fold and 2.9-fold, respectively.

## 5 Conclusion and future perspectives

Pyroptosis plays an important role in the pathogenesis of several neurodegenerative and metabolic disorders. This review examined the vicious interplay between pyroptosis, Aβ deposition, and tau pathology during AD progression, as well as the molecular mechanisms by which exercise may suppress pyroptosis. Several key conclusions can be drawn from the available evidence.

First, pyroptosis appears to be a central mechanistic link that drives the progression of AD pathology. Abnormal Aβ accumulation and tau hyperphosphorylation can act as endogenous danger signals that activate pyroptosis-related pathways, including the NLRP3/caspase-1/GSDMD axis. In turn, pyroptotic cell rupture releases pro-inflammatory mediators, such as IL-1β and IL-18, as well as ASC specks. These factors may further promote Aβ aggregation and the pathological spread of tau, thereby establishing a self-perpetuating cycle that accelerates AD progression.

Second, exercise may suppress pyroptosis through multiple, coordinated mechanisms. Exercise can promote the production of anti-inflammatory metabolites, including β-hydroxybutyrate (BHB) and lactate; activate AMPK signaling and autophagy; reduce excessive ROS accumulation; and downregulate NF-κB activity and P2 × 7 receptor expression. Through these actions, exercise may interrupt pyroptosis priming and inflammasome assembly, inhibit GSDMD cleavage and pore formation, preserve neuronal membrane integrity, and alleviate neuroinflammation.

Third, the effects of exercise are influenced by exercise modality and dose. Long-term, regular aerobic exercise and resistance training appear to consistently reduce the expression of pyroptosis-related proteins and improve cognitive outcomes. In contrast, hypoxic exercise may have a dual effect, with its consequences depending on the severity and duration of hypoxic exposure, exercise intensity, and the individual’s adaptive capacity.

Several issues warrant further investigation. First, the precise molecular mechanisms by which different exercise modalities regulate pyroptosis to prevent or alleviate AD and its complications require additional validation. Second, individualized exercise prescriptions should be developed by examining differences in exercise responses according to age, sex, and health status, as well as exercise type and intensity. Such work may help identify the most appropriate exercise strategies for different populations. Finally, clinical translation and the identification of peripheral biomarkers should be accelerated. Current evidence is derived largely from animal and cellular models. Future clinical cohort studies should evaluate whether pyroptosis-related molecules in peripheral blood, including ASC specks, microvesicles, and specific metabolites, can serve as sensitive biomarkers for monitoring pyroptosis in the AD brain and assessing the efficacy of exercise-based interventions.
